# Metformin relaxes rat thoracic aorta via nitric oxide, AMPK, potassium channels, and PKC

**DOI:** 10.22038/IJBMS.2023.69728.15179

**Published:** 2023

**Authors:** Serdar Sahinturk

**Affiliations:** 1 Bursa Uludag University Medicine School, Physiology Department, Bursa, Turkey

**Keywords:** Metformin, Nitric oxide, Potassium channels, Rat, Thoracic aorta

## Abstract

**Objective(s):**

The present research aimed to identify the functional effects and underlying mechanisms of metformin on the rat thoracic aorta.

**Materials and Methods:**

Thoracic aorta segments of Wistar Albino rats were put in the chambers of an isolated tissue bath system. The resting tone was adjusted to 1 g. Following the equilibration time, potassium chloride or phenylephrine was used to contract the vascular segments. The vessel segments were cumulatively treated with metformin (10^-7^–10^-3^ M) when a steady contraction was achieved. The described experimental approach was repeated after incubations with signaling pathway inhibitors and selective blockers of potassium channels to identify the effect mechanisms of metformin.

**Results:**

Metformin had a potent vasorelaxant effect in a concentration-dependent way (*P*<0.001). After the endothelium was removed, the vasorelaxant effect level of metformin was significantly reduced. The level of vasorelaxant effect of metformin was increased by the maintenance of perivascular adipose tissue. Following administrations of L-NAME, methylene blue, compound C, BIM-I, and potassium channel blockers, the level of vasodilatory action of metformin was significantly reduced (*P*<0.001).

**Conclusion:**

According to the results of this investigation, metformin significantly relaxes the thoracic aorta segments of rats. Metformin-mediated vasorelaxation involves the activation of numerous subtypes of potassium channels, including BKCa, IKCa, Kv, Kir, and K2p channels, as well as endothelium-dependent processes, including AMPK and eNOS/NO/sGS signaling pathways. Moreover, metformin-induced vasorelaxation is mediated through PVAT activation and the PKC signaling pathway.

## Introduction

Diabetes mellitus (DM) is one of the leading causes of morbidity and mortality worldwide. Because of the rise in obesity and the aging population, the incidence of DM is steadily rising. The most common type of DM, which has subtypes including type 1 DM, type 2 DM, and gestational DM, is type 2 DM. DM is a major risk factor for cardiovascular disorders and the main reason for mortality in DM is cardiovascular complications. Metformin (MTF) is an oral antidiabetic drug used as the first stage in the treatment of type 2 DM, even though there are numerous drug alternatives for the treatment of DM nowadays (1-7). Galega officinalis plant leaves are the source of the biguanide compound MTF. It has been used in herbal medicine for hundreds of years due to its antipyretic, analgesic, antimalarial, antiviral, and antibacterial properties. The most widely prescribed oral antidiabetic medication for diabetic people is MTF. It is a widely used medication because its insulin-sensitizing effect safely and affordably regulates blood glucose. MTF has many other beneficial effects on health in addition to its ability to treat DM, including the ability to treat preeclampsia and polycystic ovarian syndrome Furthermore, some studies suggest that MTF treatment may delay the onset of aging and cancer. 

Since the 1950s, MTF has been used to manage DM. However, there is very little information available regarding the action mechanisms of MTF. The improvement of endothelial processes is one of the most significant advantages of MTF in the management of DM. This result is crucial in avoiding cardiovascular issues brought on by DM. In this way, the complications and death linked to DM are reduced. Previous studies have highlighted the activation of AMPK (adenosine monophosphate-activated protein kinase) and increased production of nitric oxide (NO) as the mechanisms underlying this effect (8-10). However, it has not yet been thoroughly explored whether MTF-mediated vascular functional effects are mediated by mechanisms such as potassium channel activation, protein kinase C (PKC) signaling pathway, stimulation of perivascular adipose tissue (PVAT), and mitogen-activated protein kinase (MAPK) signaling pathway. The purpose of this research was to examine the vascular functional effects of MTF and the mediating mechanisms in the rat thoracic aorta (RTA). For this reason, possible roles of AMPK, MAPK, PKC, soluble guanylate cyclase (sGS), endothelial nitric oxide synthase (eNOS), prostanoids, and various potassium (K^+^) channel subtypes were questioned.

## Materials and Methods

***Ethical approval and experimental animals***

After receiving approval from the Local Animal Experiment Ethics Committee of Bursa Uludag University (2022-02/06; date: 08.02.2022), the research was launched. In the current research, 20 Wistar Albino male rats (250–300 g; 12 weeks old) served as experimental subjects. 

***Isolated tissue bath experiments***

The thoracoabdominal regions of the rats were quickly opened and their thoracic aortas were quickly removed gently. The excised thoracic aorta tissues were placed in Petri dishes containing ice-cold Krebs solution (in mM: 118 NaCl, 2.5 CaCl_2_ · 2H_2_O, 25 NaHCO_3_, 1.2 MgSO_4_ · 7H_2_O, 1.2 KH_2_PO_4_, 11 C_6_H_12_O_6_ · H_2_O, 4.8 KCl). The surrounding connective tissue and adipose tissue were meticulously removed from the thoracic aorta tissues. Four millimeter vessel segments were prepared after cleaning. Thoracic aortic segments of equal length were placed in the glass bath chambers of the 4-channel isolated tissue bath system (MAY IOBS99, Commat Ltd., Ankara, Turkey). For this procedure, steel hanging equipment and surgical sutures were used. Krebs solution was poured into the bath chambers and reservoirs. A thermal circulator was used to continuously circulate the hot distilled water in the double-jacketed system. The temperature of the Krebs solution in the bath chambers was thus held constant at 37 °C. Krebs solution was continuously gassed with a 95% O_2_–5% CO_2_ gas mixture in the bath chambers. As a result, the system’s pH was maintained at 7.4. After the first 30 min, the tone of the vascular segments was adjusted to 1 g. Every 10 min, the Krebs solution which was in contact with the tissues in the chambers was replaced. Throughout the time of equilibration, this process continued (90 min). As a result, the elimination of metabolic wastes and the nutrition of the tissues were both guaranteed. 

Phenylephrine (10^-6^ M) or potassium chloride (KCl; 60 mM) was used to induce vascular contraction following the equilibration interval. When a stable contraction was achieved, cumulative doses of MTF (10^-7^–10^-3^ M) were administered to vessel segments. In the control group, distilled water was used in place of MTF in an equivalent volume. To identify the underlying mechanisms by which MTF exhibits its vasorelaxant effects, aorta segments were treated for 30 min with potassium channel blockers or various signaling pathway inhibitors, including N-nitro-L-arginine methyl ester hydrochloride (L-NAME), bisindolylmaleimide I (BIM-I), compound C (CC), indomethacin, and methylene blue. Following that, MTF (10^-7^–10^-3^ M) was cumulatively applicated to the vascular segments that had been pre-contracted with PE during the stable contraction of the plateau period (10^-6^ M).

The perivascular adipose tissue preserved (PVAT+) vascular segment samples underwent the same experimental procedure as described above. In this way, the potential contribution of PVAT to MTF-mediated vasorelaxation was examined.

To measure the tone changes in the vessel segments, isometric force transducers (SS12LA force transducer, BIOPAC Systems, Inc. Aero Camino, USA) were employed. Computer software was used to analyze the resulting changes in vascular tone (BIOPAC MP36, Santa Barbara, CA, USA). The maximum tone value obtained with 10-6 M PE was accepted as 100%. Calculations were made over this value to determine the vascular tone variations in the thoracic aortic segments induced by administration of 10^-7^–10^-3^ M MTF.

Control group recordings for each experimental group were made at the beginning of the experiments. The same vessel segments were used to finish the experiments in the related experimental groups. The thoracic aortic segments’ equilibration and washing times were repeated for this reason.

The vascular segments with the insufficient contractile response obtained with PE (10^-6^ M) were excluded from the study. Control of endothelial integrity was done by relaxing the vessels that were precontracted with PE with acetylcholine (ACh; 10^-6^ M). A relaxation response of more than 80% was thought to be indicative of an intact endothelium (E+) (11). 

Endothelium-denuded (E-) vessel segments were used in some study groups. A wooden toothpick was used to delicately rub the inner surface of the thoracic aortic segments. Then, PE was used to constrict these vessel samples (10^-6^ M). Less than 10% of the relaxation reaction induced by ACh (10^-6^ M) indicated that the endothelium was removed. The current study’s experimental methods were all carried out following previous studies’ instructions (11-16).

***Drugs***

The substances and medications of this research were purchased from Sigma-Aldrich. The literature has established the dosages for MTF, PE, KCl, ACh, L-NAME, methylene blue, BIM-I, CC, indomethacin, and all blockers of the K^+^ channel, and the medications have been prepared following the user instructions. Distilled water was used to dissolve the following substances: PE (10^-6^ M), ACh (10^-6^ M), MTF (10^-7^–10^-3^ M), TEA (10 mM), L-NAME (10^-4^ M), apamin (100 nM), methylene blue (10^-5^ M), iberiotoxin (20 nM), BaCl_2_ (30 µM), 4-AP (1 mM), and TRAM-34 (18 nM). In dimethyl sulfoxide, the following substances were dissolved: CC (10 µM), BIM-I (150 nM), U0126 (1.5 µM), indomethacin (5 µM), glyburide (10 M), and anandamide (10 µM) (DMSO). The ultimate DMSO concentration in the Krebs solution was less than 0.1%. The reactions of vascular smooth muscle (VSM) to contraction or relaxation were unaffected by DMSO.

***Statistical analysis***

A power analysis was conducted before the research began, and the minimum sample size was determined. The obtained data were statistically analyzed using SPSS v.23.0 (SPSS Inc., Chicago, IL, USA). The information was displayed as mean±standard deviation. “n” represents the total number of vessel segments used across all study groups. MTF-induced relaxation responses were expressed as a percentage of the maximal plateau period contraction brought on by PE (10^-6^ M). A one-way ANOVA test followed by Bonferroni *post hoc* test was used to identify statistical differences between multiple groups.

## Results

***The vasorelaxant effect of MTF in the RTA segments***

**Figure 1:**
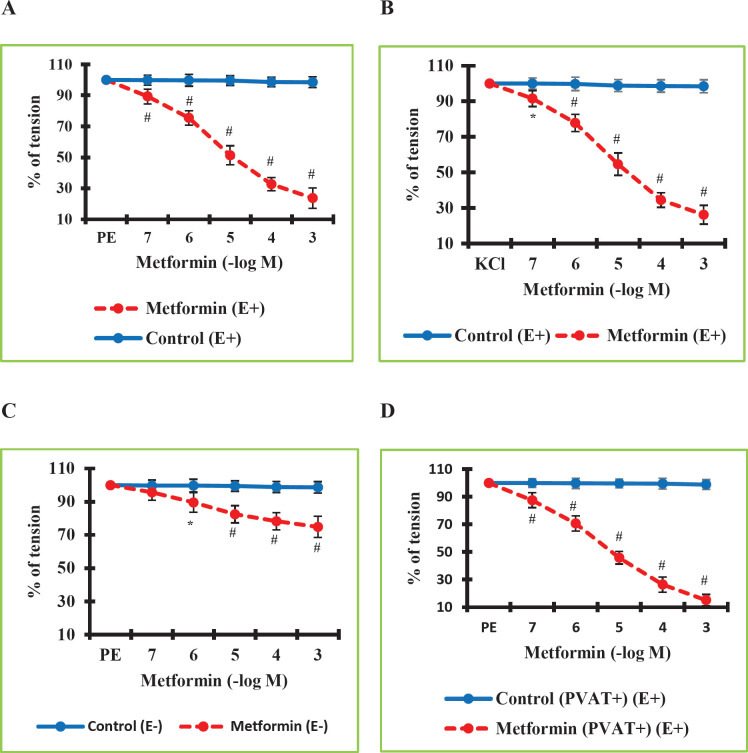
A. Metformin-induced vasorelaxant activity in the rat thoracic aorta precontracted with PE. Percentage tension values in the Metformin (E+) group were found significantly lower than in the Control (E+) group. B. Metformin-induced vasorelaxant activity in the rat thoracic aorta precontracted with KCl. Percentage tension values in the Metformin (E+) group were found significantly lower than in the Control (E+) group. C. Effect of removal of the endothelium on the vasorelaxant activity of metformin. Percentage tension values in the Control endothelium-denuded (E-) group were found significantly higher than in the Metformin (E-) group. D. Effect of preserving perivascular adipose tissue (PVAT) on the vasorelaxant activity of Metformin in the (E+) rat thoracic aortic rings precontracted with phenylephrine. Percentage tension values in the Metformin (PVAT+) (E+) group were found lower than in the Control (PVAT+) (E+) group. The data were expressed as mean ± standard deviation as a percentage of the phenylephrine-induced plateau tension. n = 8 in each group

The initial stage of our study focused on determining the vasodilatory effect of MTF. For this purpose, after the vascular segments were contracted with PE or KCl, increasing concentrations of MTF were administered in the plateau phase. The calculated values were compared with those of the control group. The values in the MTF (E+) group were found to be significantly lower than the values in the Control (E+) group (*P*=0.000 at a concentration of 10^-6^ M). MTF had a significant vascular relaxation effect in RTA (E+). The highest relaxation rate was about 76.23%. (Figure 1A). Additionally, MTF induced significant vascular relaxation when KCl was used to pre-contract RTA (E+) (*P*=0.000 at a concentration of 10^-3^ M). The highest relaxation rate was around 73.82% (Figure 1B). These findings revealed that MTF significantly reduced PE- and KCl-mediated vascular constriction in the RTA (E+).

***Participation of endothelium in the vasorelaxant effect of MTF on the RTA segments***

In the second stage of our study, it was questioned whether endothelium was involved in the vasorelaxant effect of MTF. To this end, after the endothelium-removed vascular segments were contracted with PE, increasing concentrations of MTF were administered in the plateau phase. The calculated values were compared with those of the control group. The determined values in the MTF (E-) group were found to be significantly lower than the values in the Control (E-) group (*P*=0.000 at a concentration of 10^-3^ M). Due to the removal of the vascular endothelium, the level of MTF-induced vascular relaxation was greatly decreased, down to about 25.16% (Figure 1C). These results demonstrated the involvement of endothelium-independent mechanisms in MTF-induced vasodilation in addition to endothelium-dependent mechanisms. Vascular relaxation induced by MTF is greatly reduced by the removal of the endothelium but is not eliminated. These results indicated that mechanisms that are endothelium-dependent play a larger part in MTF-mediated vasodilation.

***Participation of PVAT in the vasorelaxant effect of MTF on the RTA segments***

In the third phase of our study, it was questioned whether PVAT contributed to MTF-induced vasorelaxation. For this purpose, after the PVAT-preserved vascular segments were contracted with PE, increasing concentrations of MTF were administered in the plateau phase. The calculated values were compared with those of the control group. The determined values in the MTF (PVAT+) (E+) group were observed to be significantly lower than the values in the Control (PVAT+) (E+) group (*P*=0.000 at a concentration of 10^-3^ M). By preserving the vascular PVAT, the vasorelaxant effect of MTF was slightly increased, hitting a level of about 84.96% (Figure 1D). This result suggested that PVAT-mediated mechanisms may be involved in MTF-induced vasodilation.

***Participation of the eNOS/NO/sGS signaling pathway in the vasorelaxant effect of MTF on the RTA segments***

**Figure 2:**
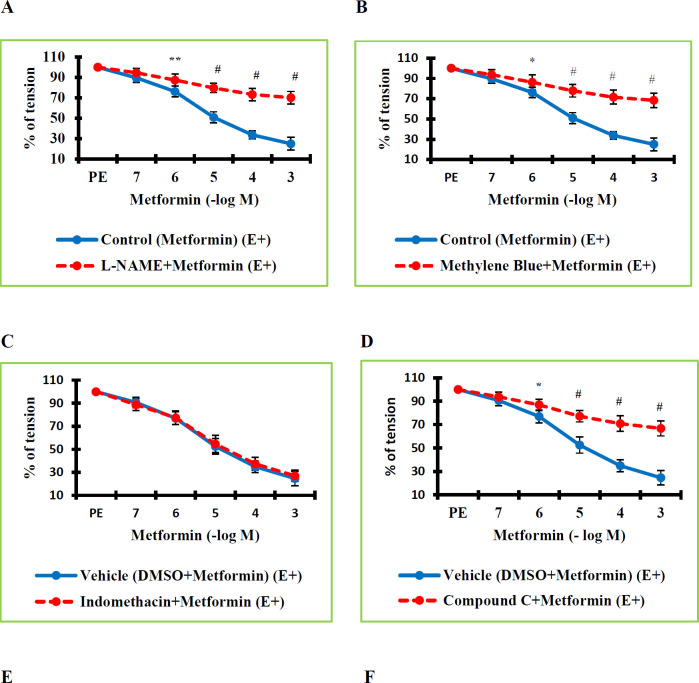
A. Effect of endothelial nitric oxide synthase (eNOS) inhibition on the vasorelaxant effect of metformin. Percentage tension values in the L-NAME+Metformin (E+) group were found statistically significantly higher than in the Control (Metformin) (E+) group. B. Effect of soluble guanylate cyclase (sGS) inhibition on the vasorelaxant effect of metformin. Percentage tension values in the Methylene Blue+Metformin (E+) group were found statistically significantly higher than in the Control (Metformin) (E+) group. C. Effect of cyclooxygenase (COX) 1/2 inhibition on the vasorelaxant activity of metformin. Percentage tension values in the Indomethacin+Metformin (E+) group were found significantly higher than in the Vehicle (DMSO+Metformin) (E+) group. D. Effect of adenosine monophosphate-activated protein kinase (AMPK) inhibition on the vasorelaxant activity of metformin. Percentage tension values in the Compound C+Metformin (E+) group were found significantly higher than in the Vehicle (DMSO+Metformin) (E+) group. E. Effect of protein kinase C (PKC) inhibition on the vasorelaxant activity of metformin. Percentage tension values in the BIM-I+Metformin (E+) group were found significantly higher than in the Vehicle (DMSO+Metformin) (E+) group. F. Effect of mitogen-activated protein kinase (MAPK)/extracellular signal-regulated kinase (ERK) 1/2 inhibition on the vasorelaxant activity of metformin. Percentage tension values in the U0126+Metformin (E+) group were found significantly higher than in the Vehicle (DMSO+Metformin) (E+) group. The data were expressed as mean ± standard deviation as a percentage of the phenylephrine-induced plateau tension. n = 8 in each group

In the final stage of our investigation, we determined the processes underlying the vasorelaxant effects of MTF. This is why we began by focusing on mechanisms involving the endothelium. To this end, after the equilibration period, 30 min of L-NAME and methylene blue incubations were administered to the vessels. Then, increasing concentrations of MTF were applied to the vessel segments in the plateau phase that were precontracted with PE. The calculated values were compared with those of the control group. The detected values in the L-NAME+MTF (E+) group were determined to be significantly greater than the values in the Control (MTF) (E+) group (*P*=0.007 at a concentration of 10^-6^ M; *P*=0.000 at concentrations of 10^-5^–10^-3^ M). Due to eNOS inhibition, the value of the vasorelaxant effect of MTF in the RTA was significantly decreased (Figure 2A). The determined values in the Methylene Blue+MTF (E+) group were found to be significantly higher than the values in the Control (MTF) (E+) group (*P*=0.028 at a concentration of 10^-6^ M; *P*=0.000 at concentrations of 10^-5^–10^-3^ M). Due to sGS inhibition, the vasorelaxant effect level of MTF in the RTA was significantly diminished (Figure 2B). These findings proved that the eNOS/NO/sGS signaling pathway plays a significant part in the vasodilatory action of MTF.

***Participation of the COX signaling pathway in the vasorelaxant effect of MTF in the RTA segments***

We questioned whether prostanoids, a different endothelium-related mechanism, were involved in MTF-induced vasorelaxation. A 30 min indomethacin incubation was applied to the vessels to inhibit the COX signaling pathway. Then, plateau tone was obtained by contracting the vessels with PE. Increasing concentrations of MTF were administered. The calculated values were compared with those of the vehicle group. There were no significant differences in the tone values between the Indomethacin+MTF (E+) group and the Vehicle (DMSO+MTF) (E+) group (*P*=1.000 at concentrations of 10^-7^–10^-3^ M). Non-selective COX inhibition did not significantly change the magnitude of the vasodilator effect of MTF (Figure 2C). This finding indicated that the vasorelaxation mediated by MTF is not related to prostanoids.

***Association of MTF-induced vasorelaxation with other possible signaling pathways in the RTA***

We used CC as an AMPK inhibitor to determine whether the AMPK signaling pathway, which is responsible for many metabolic effects of MTF, is implicated in MTF-induced vasorelaxation. After 30 min of incubation with CC, vessel samples were contracted with PE. Increasing concentrations of MTF were administered to the vessels in which a stable contraction was achieved. The calculated values were compared with those of the vehicle group. The percentage tone values in the CC+MTF (E+) group were found to be significantly greater than those in the Vehicle (DMSO+MTF) (E+) group. Inhibiting AMPK significantly decreased the amount of MTF’s vasodilator effect (*P*=0.016 at a concentration of 10^-6^ M; *P*=0.000 at a concentration of 10^-5^–10^-3^ M) (Figure 2D). This finding demonstrated that the AMPK signaling pathway mediates both the metabolic and vasodilator effects of MTF.

The contribution of PKC signaling to the vasodilatory effect of MTF was questioned. To this end, after 30 min of incubation with BIM-I, vessel samples were contracted with PE. Increasing concentrations of MTF were administered to the vessels in which a stable contraction was achieved. The calculated values were compared with those of the vehicle group. The percentage tone values in the BIM-I+MTF (E+) group were found to be significantly greater than those in the Vehicle (DMSO+MTF) (E+) group. PKC inhibition substantially decreased the level of vasodilator effect that MTF had (*P*=0.040 at a concentration of 10^-6^; *P*=0.000 at concentrations of 10^-5^–10^-3^ M) (Figure 2E). These results implied that the PKC signaling pathway, which controls many cellular processes, plays a role in the vasorelaxant activity of MTF.

The contribution of MAPK signaling to the vasodilatory effect of MTF was questioned. To this end, after 30 min of incubation with U0126, vessel samples were contracted with PE. Increasing concentrations of MTF were administered to the vessels in which a stable contraction was achieved. The calculated values were compared with those of the vehicle group. There was no statistically significant difference in the percentage tone values between the U0126+MTF (E+) group and the Vehicle (DMSO+MTF) (E+) group (*P*>0.05). Inhibition of MAPK did not affect the vasodilator effect of MTF (Figure 2F). This finding proved that the MAPK signaling pathway is not involved in MTF-mediated vascular relaxation.

***Role of potassium channels in the vasorelaxant effect of MTF in the RTA segments***

**Figure 3:**
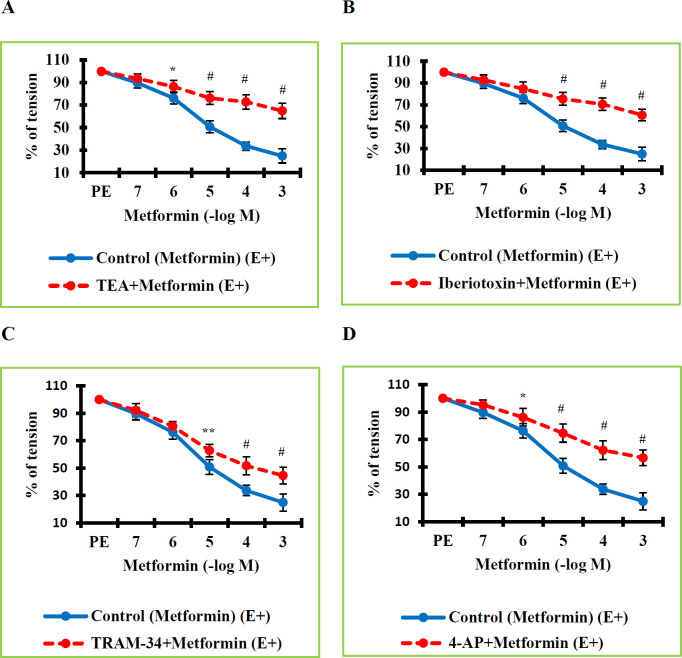
A. Effect of non-selective K+ channel blocking on the vasorelaxant effect of metformin. Percentage tension values in the TEA+Metformin (E+) group were found statistically significantly higher than in the Control (Metformin) (E+) group. B. Effect of large-conductance Ca^2+^-activated K+ (BKCa) channel blocking on the vasorelaxant activity of metformin. Percentage tension values in the Iberiotoxin+Metformin (E+) group were found significantly higher than in the Control (Metformin) (E+) group. C. Effect of intermediate-conductance Ca^2+^-activated K+ (IKCa) channel blocking on the vasorelaxant activity of metformin. Percentage tension values in the TRAM-34+Metformin (E+) group were found significantly higher than in the Control (Metformin) (E+) group. D. Effect of voltage-gated K+ (Kv) channel blocking on the vasorelaxant effect of metformin. Percentage tension values in the 4-AP+Metformin (E+) group were found statistically significantly higher than in the Control (Metformin) (E+) group. n = 8 in each group

**Figure 4:**
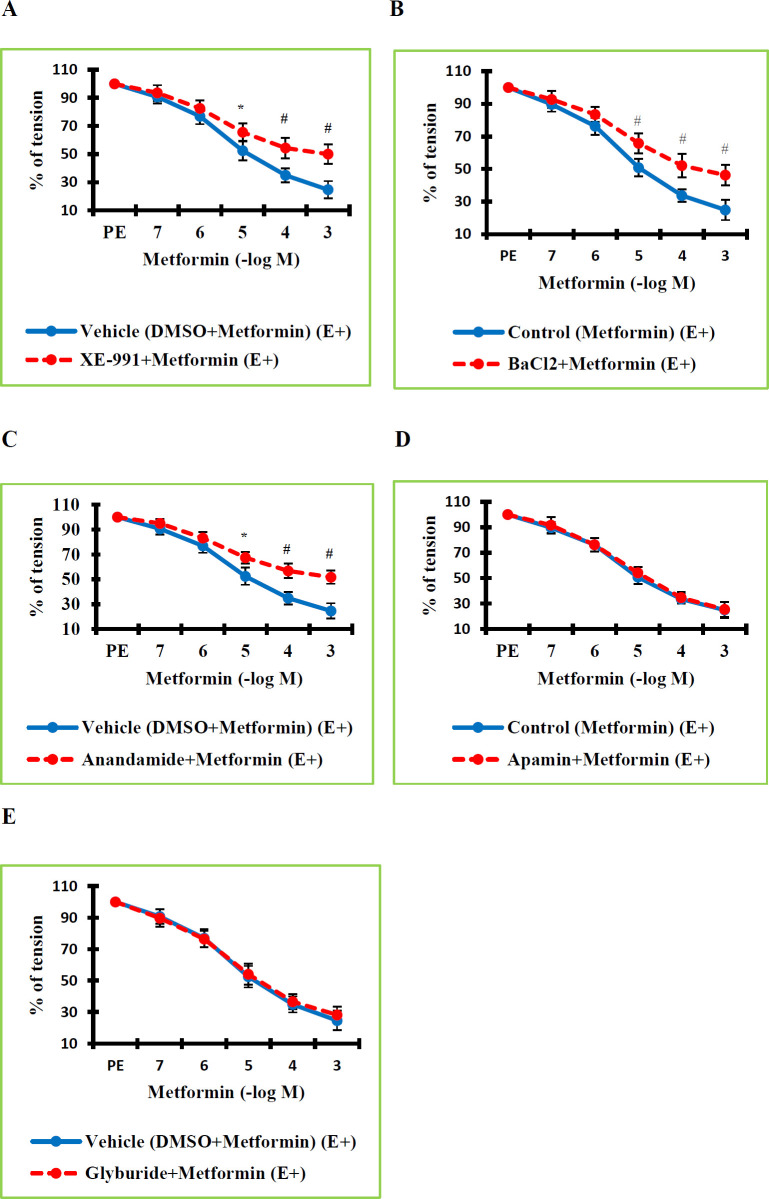
A. Effect of Kv7.1-7.5 channel blocking on the vasorelaxant activity of metformin in the endothelium-intact (E+) rat thoracic aortic rings precontracted with phenylephrine. Percentage tension values in the XE-991+Metformin (E+) group were found significantly higher than in the Vehicle (DMSO+Metformin) (E+) group. B. Effect of inward-rectifier K+ (Kir) channel blocking on the vasorelaxant effect of metformin. Percentage tension values in the BaCl2+Metformin (E+) group were found statistically significantly higher than in the Metformin (E+) group. C. Effect of TASK-1, a two-pore domain K+ (K2P) channel, blockage on the vasorelaxant effect of metformin. Percentage tension values in the Anandamide+Metformin (E+) group were found statistically significantly higher than in the Vehicle (DMSO+Metformin) (E+) group. D. Effect of small conductance Ca^2+^-activated K+ (SKCa) channel blocking on the vasorelaxant effect of metformin. There was no statistically significant difference between the percent tension values of the Apamin+Metformin (E+) group and the Control (Metformin) (E+) group. E. Effect of ATP-sensitive K+ (KATP) channel blocking on the vasorelaxant effect of metformin. Percentage tension values in the Glyburide+Metformin (E+) group were found statistically significantly higher than in the Vehicle (DMSO+Metformin) (E+) group. n = 8 in each group

The final step of our research was to investigate whether the mechanisms underlying the vasorelaxant effects of MTF include K^+^ channels. For this purpose, we employed both non-selective and selective potassium channel blockers. A 30-minute incubation was performed with potassium channel blockers. MTF was administered in increasing concentrations to vessels contracted by PE during the plateau phase. The calculated values were compared with those of the control or vehicle group. The tone values in the TEA+MTF (E+) group were determined to be significantly higher than the values in the Control (MTF) (E+) group (*P*=0.023 at a concentration of 10^-6^ M; *P*=0.000 at concentrations of 10^-5^–10^-3^ M) (Figure 3A). As TEA is not a selective potassium channel blocker, it was necessary to demonstrate which subtypes of potassium channels are related to the vasodilating effect of MTF, even if these findings suggest that K^+^ channels have a role in MTF-mediated vascular relaxation. Then, we determined which K^+^ channel classes are responsible for MTF-induced vasodilation using selective K^+^ channel blockers. BK_Ca_ channels were selectively blocked by 30 min of iberiotoxin incubation. Afterward, increasing concentrations of MTF were applied to the vessels contracted with PE. The calculated values in the Iberiotoxin (a BK_Ca _channel blocker)+MTF (E+) group were determined to be significantly higher than the values in the Control (MTF) (E+) group (*P*=0.000 at concentrations of 10^-5^–10^-3^ M) (Figure 3B). IK_Ca_ channels were selectively blocked by 30 min of TRAM-34 incubation. The calculated values in the TRAM-34 (an IK_Ca_ channel blocker)+MTF (E+) group were determined to be significantly higher than the values in the Control (MTF) (E+) group (*P*=0.002 at a concentration of 10^-5^ M; *P*=0.000 at concentrations of 10^-4^–10^-3^ M) (Figure 3C). K_V_ channels were blocked by 30 min of 4-AP incubation. The values in the 4-AP (a Kv channel blocker)+MTF (E+) group were found to be significantly higher than the values in the Control (MTF) (E+) group (*P*=0.030 at a concentration of 10^-6^ M; *P*=0.000 at concentrations of 10^-5^–10^-3^ M) (Figure 3D). Kv7.1-7.5 channels were selectively blocked by 30 min of XE-991 incubation. The values for the XE-991 (a Kv7.1-7.5 channel blocker)+MTF (E+) group were determined to be significantly higher than the values in the Vehicle (DMSO+MTF) (E+) group (*P*=0.001 at a concentration of 10^-5^ M; *P*=0.000 at concentrations of 10^-4^–10^-3^ M) (Figure 4A). Kir channels were selectively blocked by 30 min of BaCl_2_ incubation. The values in the BaCl_2_ (a Kir channel blocker)+MTF (E+) group were determined to be significantly higher than the values in the Control (MTF) (E+) group (*P*=0.000 at concentrations of 10^-5^– 10^-3^ M) (Figure 4B). K_2p_ TASK-1 channels were selectively blocked by 30 min of XE-991 incubation. The values in the Anandamide (a K_2p_ TASK-1 channel blocker)+MTF (E+) group were determined to be significantly higher than the percentage tone values in the Vehicle (DMSO+MTF) (E+) group (*P*=0.001 at a concentration of 10^-5^ M; *P*=0.000 at concentrations of 10^-4^–10^-3^ M) (Figure 4C). Due to blocking BK_Ca_, IK_Ca_, Kv, Kv7.1-7.5, K_ATP_, K_2p_, and Kir channels, the level of MTF that had a vasorelaxant effect in the RTA was significantly decreased. These findings demonstrated that BK_Ca_, IK_Ca_, Kv, Kv7.1-7.5, K_2p_ TASK-1, and Kir channels participate in MTF-induced vasorelaxation.

K_ATP_ and SK_Ca_ channels were selectively blocked by 30 min of glyburide and apamin incubations. There was no significant difference between the tone values in the Apamin (an SK_Ca_ channel blocker)+MTF (E+) group and the Control (MTF) (E+) group (*P*=1.000 at concentrations of 10^-7^–10^-3^ M) (Figure 4D). Additionally, there was no significant difference between the tone values in the Glyburide (a K_ATP_ channel blocker)+MTF (E+) group and the Vehicle (DMSO+MTF) (E+) group (p=1.000 at concentrations of 10^-7^–10^-3^ M) (Figure 4E). Following SK_Ca_ or K_ATP_ channel blockade, there was no significant change in the vasorelaxant effect level of MTF in the RTA. These results implied that the SK_Ca_ and K_ATP_ channels are not involved in the mechanisms underlying the vasorelaxant effects of MTF.

*: *P*< 0.01. #:*P*< 0.001. E+: endothelium intact. E-: endothelium denuded. KCl: potassium chloride. PE: phenylephrine

*:*P*< 0.05. **: *P*< 0.01. #: *P*< 0.001. E+: endothelium intact. PE: phenylephrine

*: *P*< 0.05. **:*P*< 0.01. #: *P*< 0.001. E+: endothelium intact. PE: phenylephrine

*: *P*< 0.01. #: *P*< 0.001. E+: endothelium intact. PE: phenylephrine

## Discussion

MTF, which is an effective drug that provides significant benefits in the treatment of DM, still maintains its importance today, as obesity and DM increase as an epidemic. The French doctor Jean Sterne published the first study on the use of MTF in the treatment of DM in 1957 (17). Its use was discontinued for a while, due to its relatively low blood sugar-lowering effect and causing lactic acidosis. It soon regained its well-deserved reputation for having many beneficial effects. These are features such as preventing weight gain, overcoming insulin resistance, and not causing an increase in the risk of hypoglycemia. The use of MTF, which is gaining confidence due to these beneficial properties, was approved in the USA in 1995. The first step medication used to treat type 2 DM, which is becoming more and more common and has reached epidemic proportions, is MTF. In addition, due to the sensitizing effect of insulin and preventing weight gain, MTF is also used in the treatment of diseases such as obesity, polycystic ovary syndrome, and type 1 DM (1, 2, 18). Despite being crucial in the management of DM, MTF’s cellular effect mechanisms of action are still inadequately understood. MTF treatment is associated with vascular relaxation and a decrease in blood pressure, according to studies in the literature (10, 18). However, it is unknown how these effects occur. Since type 2 DM is a very important risk of cardiovascular disease and can cause complications such as heart failure and hypertension, understanding the mechanisms of cardiovascular action is very important in terms of understanding how the use of MTF can provide benefits against these pathologies. Therefore, in our study, the vasoactive effects and mechanisms of action of MTF in the RTA model were investigated. Our study revealed that MTF has a vasorelaxant effect. MTF causes vasorelaxation by activation of complex mechanisms, and many of these mechanisms were demonstrated for the first time in this study. It has been determined that endothelium-dependent, PVAT-related, and endothelium-independent mechanisms have a role in the formation of this effect. The eNOS/NO, AMPK, MAPK, and PKC signaling pathways have been observed to be involved in the vasorelaxant effect of MTF. Moreover, PVAT, prostanoids, and many subtypes of K^+ ^channels have been shown to contribute to MTF-induced vasorelaxation.

In previous studies, the functional effects of MTF administration in various smooth muscle tissues such as the uterus, ileum, and corpus cavernosum have been investigated. Hehir and Morrison (19) reported that MTF did not exert any relaxant effect on spontaneous or oxytocin-induced contractions of human uterine smooth muscle. In one study (20), a similar experimental protocol was applied to the uterine smooth muscle of pregnant and non-pregnant rats. Those researchers found that MTF did not have a significant effect on uterine contractions. Another study demonstrated that MTF administration increased adenosine-induced relaxation in the rabbit corpus cavernosum. In that study, it was suggested that MTF increases adenosine signaling and NO production (21). In a recent study (22), it was determined that MTF causes smooth muscle relaxation in the rat bladder. Those studies demonstrated that the smooth muscle relaxant effect of MTF exists in many different tissues. Consequently, these data suggest that MTF treatment may be beneficial in pathological conditions where smooth muscles exhibit abnormal contraction.

The first finding of our study is that MTF, administered cumulatively at concentrations of 10^-7^–10^-3^ M, significantly caused vasorelaxation in the RTA. The RTA model is proposed as the gold standard model for investigating the mechanisms of action of the drugs which have antihypertensive and vasorelaxant effect potential (23). Therefore, thoracic aorta segments were used in our study. There are very few studies examining MTF’s effects and associated processes in vascular tissues. In the studies using rat tail artery samples, it was determined that MTF administration significantly reduced KCl, norepinephrine, PE, 5-hydroxytryptamine, and arginine vasopressin-induced contractions. Those studies demonstrated that MTF induces vasorelaxation in both endothelium-intact and -removed rat tail arteries (24-26). On the other hand, a few other studies have used rat aorta segments. In those studies, besides a vasorelaxant effect similar to previous studies, the researchers found that the AMPK and eNOS/NO signaling pathways have a crucial role in this effect of MTF (8-10). The results of our study supported the findings of previous studies and showed that MTF causes vasorelaxation in a concentration-dependent manner in the RTA with intact endothelium. The maximal vasorelaxant effect level of MTF was found quite high (approximately 77.33%). 

In the continuation of our study, we aimed to investigate the mechanisms of MTF-mediated vasorelaxation. For this purpose, the level of contribution of endothelium to the vasorelaxation induced by MTF and whether PVAT contributed or not were investigated. Our data indicate that MTF-mediated vasorelaxation occurs mostly by endothelium-dependent mechanisms. However, the removal of the endothelium did not completely abolish the vasorelaxant effect of MTF. The maximal vasorelaxant effect level of MTF at the maximal concentration was found to be approximately 25.16% in endothelium-denuded thoracic aorta segments. Consequently, endothelium-independent mechanisms may also contribute to MTF-induced vasorelaxation. On the other hand, it is known that many mediators such as apelin, leptin, and nesfatin secreted by PVAT can cause antihypertensive and vasorelaxant effects. In addition, it has been suggested that PVAT-derived mediators are closely associated with AMPK activation (27). Therefore, in our study, the effect of MTF was also investigated in PVAT-preserved vessel samples, considering that PVAT-related mechanisms may have a role in the vasorelaxant effect of MTF. It was determined that PVAT preserving provided a slight increase in the level of vasorelaxant effect caused by MTF. In these vessel samples, the maximal vasorelaxant effect level of MTF was observed as approximately 84.96%. These data suggested that MTF may stimulate the secretion of various vasorelaxant-acting mediators from PVAT.

In the next step, due to the endothelium-dependent vasorelaxant effect of MTF, the relationship between MTF-induced vasorelaxation and endothelium-dependent mechanisms was questioned. It is known that the eNOS/NO/sGS signaling pathway is the most effective mechanism that mediates endothelium-dependent vasorelaxation. Various mediators cause an increase in eNOS activity, followed by an increase in NO production in endothelial cells. NO diffuses into VSM cells (VSMCs) and binds to the sGS receptor in the cytoplasm, activating mechanisms mediating vasodilation (28). The results of our study showed that the eNOS inhibitor L-NAME and the sGS inhibitor methylene blue applications significantly and greatly reduce the vasorelaxant effect levels of MTF. These data pointed to the importance of the eNOS/NO/sGS signaling pathway in MTF-induced vasorelaxation. On the other hand, it is known that prostaglandins, especially prostaglandin I_2_, are another important endothelium-derived vasodilator mechanism (29). Therefore, we looked into whether the non-selective COX 1/2 inhibitor indomethacin administration altered the vasorelaxant effects of MTF. It was determined that the administration of indomethacin had no discernible effect on MTF-induced vasorelaxation. As a result, our findings demonstrated that MTF has a vasodilator effect that is endothelium-dependent but unrelated to prostaglandins. Our results confirmed that the eNOS/NO/sGS signaling pathway is an important endothelium-dependent mechanism involved in the vasorelaxant mechanism of action of MTF. Similar findings were obtained in a previous study by Majithiya and Balaraman (8). In that study, using thoracic aorta segments obtained from diabetic and non-diabetic rats, the vasorelaxant effect of MTF was blocked by the removal of the endothelium or administration of L-NAME. Methylene blue had a comparable result. On the other hand, there was no remarkable difference in the level of MTF’s vasorelaxant effect following the administration of indomethacin. Consequently, they concluded that eNOS/NO/sGS signaling pathway plays a role in MTF-induced vasorelaxation. However, there was no contribution of prostanoids. The findings obtained in the present study were consistent with the results of Majithiya and Balaraman’s study. However, in the present study, healthy rat aortas were used, not diabetic rat aortas. Consequently, all these data demonstrate that MTF-induced vascular relaxation is associated with eNOS/NO/sGS signaling pathway in physiological conditions as well as in pathological conditions. On the other hand, prostanoids do not contribute to MTF-induced vasorelaxation.

It is known that the AMPK signaling pathway is associated with VSM contraction and relaxation functions. Vasodilator and hypotensive effects have been reported in various studies using AMPK activators such as AICAR or A769662 (30, 31). Researchers (32) reported that AMPK is involved in endothelium-independent vasorelaxation in the mouse aorta. Vasorelaxation may occur as a result of mechanisms such as activated potassium channels after the activated AMPK signaling pathway or the eNOS/NO/sGS signaling pathway. The metabolic effects of MTF in liver hepatocytes, the main site of action, are mediated by AMPK activation. This is the underlying mechanism by which MTF reduces glucose production and fatty acid oxidation (33). On the other hand, the AMPK signaling pathway plays a role in MTF’s effects on increasing eNOS phosphorylation and NO bioavailability, as well as reducing VSM cell proliferation, migration, and inflammatory responses (24, 35). In previous studies, it was determined that MTF administration increased the expression of AMPK in vascular tissues such as the thoracic aorta. Activation of the LKB1 (Liver Kinase B1) signaling pathway has been reported to be the mechanism that stimulates AMPK activation (9, 10). Researchers (9) determined that a 30-minute incubation of MTF significantly reduced PE-induced contraction in RTA. The study, which showed that AMPK inhibitor CC application inhibited this effect of MTF, revealed that AMPK expression in VSMCs increased with MTF incubation. The researchers have reported that the AMPK signaling pathway plays a role in MTF-mediated vascular relaxation. Pyla *et al*. (10) reported that there was no significant change in PE-induced rat aorta contraction after 30 min of MTF incubation. In contrast, the researchers determined that PE-induced aorta contraction was significantly inhibited after 2 hr of MTF incubation. The researchers who showed that CC incubation inhibited this effect of MTF found an increase in AMPK expression. Therefore, it was concluded that the vasorelaxant effect of MTF was related to AMPK. In the present study, MTF was applied to the contracted RTA with PE and it was determined that it has a relaxing effect. It was shown that the vasorelaxant effect of MTF appeared in a short time. Furthermore, consistent with the study of Pyla *et al*., CC administration inhibited the vasorelaxant effect of MTF. The maximal vasorelaxant effect level of MTF in this experimental group was determined as approximately 34.32%. Therefore, in addition to its role in both other smooth muscle functions and energy metabolism, the findings indicate that the AMPK signaling pathway is an important mediator in the vasorelaxant effect of MTF in the RTA. Mechanisms such as the stimulation of the eNOS/NO signaling pathway and potassium channel activation after the AMPK signaling pathway may be inducing MTF-mediated vasorelaxation.

Numerous other mechanisms may also be involved in the vasorelaxation effect of MTF, despite studies in the literature that point to AMPK and eNOS/NO signaling pathways as potential mediators. These include mechanisms such as potassium channel activation, as well as PKC and MAPK signaling pathways. The MAPK signaling activation reveals a crucial connection between the contractile and relaxing processes of the heart. According to studies, the porcine palmar lateral vein and the ferret aorta’s contraction and relaxation responses are regulated by an extracellular signal-regulated kinase (ERK) 1/2, which is triggered in the MAPK signaling pathway (36-38). Particularly concerning the impacts of the apelinergic system on cardiovascular contractile functions, the MAPK/ERK 1/2 signaling pathway is a crucial regulator (39). The MAPK/ERK 1/2 signaling and the vasoactive effects of MTF have not been linked in any studies to date. In our research, it was found that the application of the ERK 1/2 inhibitor U0126 did not significantly alter the level of MTF’s vasorelaxant effect. Our findings suggest that the MAPK/ERK 1/2 signaling pathway does not contribute to the vasorelaxant effect of MTF.

The regulation of smooth muscle contraction and vascular functions may be influenced by mechanisms connected to the PKC signaling pathway, which is involved in numerous cellular processes. PKC can induce vascular contraction by making myofilaments more sensitive to Ca^2+^, which leads to increased vascular contraction. eNOS/NO signaling can be activated or inhibited by PKC, regulating VSM contraction and relaxation. PKC is found in a range of vascular beds in various isoforms. The different functional effects of these isoforms are thought to be responsible for PKC’s conflicting effects on vascular functions (40). Similar to the present study, Demirel *et al*. (41) recently determined that the PKC signaling pathway contributes to the vasorelaxant effect of irisin in RTA. In that study, BIM-I was used for PKC inhibition, and it was observed that the relaxant effect of irisin on vessel segments contracted by PE decreased significantly. These data demonstrate the important role of the PKC signaling pathway in vascular functional effects. To date, studies have not found a link between the hypotensive or vasorelaxant mechanism of action of MTF and the PKC signaling pathway. However, the current study determined that when the PKC signaling pathway was inhibited by BIM-I, there was a significant decrease in the vasorelaxant effect level of MTF. This finding demonstrates that the PKC signaling pathway contributes to MTF-induced vasorelaxation.

A crucial role for potassium channels in the contraction and relaxation processes of VSM has been suggested by earlier functional studies (13-16). VSMCs hyperpolarize as a result of the potassium channels in those cells being activated, which causes the cells to relax. A rise in intracellular Ca^2+^ leads to the contraction of smooth muscle in the arteries. The interaction and creation of cross-bridges between actin and myosin follow the rise in intracellular Ca^2+ ^concentration. A vital regulator of the membrane potential and vascular tone of VSMCs is the physiological activity of different potassium channels, particularly voltage-gated potassium channels. When it comes to controlling how L-type calcium channels open and close, Kv channel activity is a crucial component. Kv channels thus significantly influence the level of cytoplasmic calcium. Voltage-gated Ca^2+^ channels are inhibited after Kv channels are activated. As a result, VSMCs hyperpolarize, Ca^2+ ^influx decreases and VSM relaxes. However, blocking Kv channels results in the opposite process, which causes VSM to contract (42, 43). Previous studies have shown that many peptide and nonpeptide mediators induce vasorelaxation by activating potassium channels. A study (44) determined that diosmetin-induced vasodilation was significantly reduced after incubation with potassium channel blockers such as TEA and 4-AP, and potassium channel activation was involved in diosmetin-mediated vascular relaxation. Research (23) determined that various potassium channels such as Kv, Kir, K_ATP_, and BK_Ca_ play a role in the vasorelaxant effect of resveratrol. Demirel et al. (16, 45) determined that Kv, Kir, K_ATP_, BK_Ca_, IK_Ca_, and K_2P _channels have a role in the vasorelaxant effect of irisin. In the studies of Sahinturk et al. (13-15) and another study (46), it was found that apelin and elabela-induced vasodilation occurs by activating potassium channels such as Kv, Kir, K_ATP_, BK_Ca_, and IK_Ca_. In those studies, it was concluded that incubation of potassium channel blockers significantly reduced vascular relaxation caused by peptide agents such as apelin, elabela, and irisin. Those data suggest that potassium channel activation may contribute to the vascular functional effects of MTF, a peptide drug. As a result, in this phase of the research, we looked into the potential contribution of potassium channel subtypes that have been implicated in VSM to MTF-induced vasorelaxation responses. We used both non-selective and selective potassium channel blockers for this reason. Following the application of TEA, iberiotoxin, 4-AP, BaCl_2_, TRAM-34, anandamide, and XE-991, the vasorelaxant effect level of MTF significantly decreased. On the other hand, the MTF-induced vascular relaxation did not significantly alter following the administration of apamin and glyburide. These findings revealed that the vasorelaxation brought on by MTF involves BK_Ca_, IK_Ca_, Kv, Kir, and K_2p_ channels. However, SK_Ca_ and K_ATP _channels are not involved in MTF-mediated vasorelaxant effects. 

Our study, along with previous data, suggests that MTF may have beneficial effects on cardiovascular diseases, particularly in the treatment of hypertension, by promoting vasodilation and improving endothelial functions. Several studies in the literature support this claim. For instance, Majithiya and Balaraman (3) reported that MTF administration improved endothelial function and reduced blood pressure in streptozotocin-induced diabetic rats. Another study found that MTF administration in spontaneously hypertensive rats decreased blood pressure and attenuated the agonist-induced Ca^2+^ response in VSMCs (47). These findings suggest that MTF treatment, particularly in individuals with hypertension, may help lower arterial blood pressure (48).

## Conclusion

The present research demonstrates that MTF has vasorelaxant effects on the RTA, and it acts through multiple mechanisms. MTF produces significant vasodilation by activating both endothelium-dependent and -independent mechanisms. The vascular relaxation effect of MTF is further enhanced by PVAT. MTF-induced vasorelaxation is mediated by the PKC signaling pathway and several potassium channel subtypes, including BK_Ca_, IK_Ca_, K_V_, K_2p_, and Kir channels. Additionally, endothelium-dependent mechanisms involving AMPK and the eNOS/NO/sGS signaling pathways contribute to MTF-induced vasorelaxation. These findings suggest that MTF may be a promising treatment for hypertensive diseases associated with increased vasoreactivity. The results of this study provide insights for future research on the clinical use of MTF.

## Authors’ Contributions

All of this article was prepared and written by Serdar Şahintürk.

## Funding

No financial support has been received. 

## Conflicts of Interest

There are no conflicts of interest. 
